# Label free quantitative proteomic analysis reveals the physiological and biochemical responses of *Arabidopsis thaliana* to cinnamon essential oil

**DOI:** 10.1038/s41598-025-89368-4

**Published:** 2025-02-20

**Authors:** Sofiene Ben Kaab, Manon Martin, Hervé Degand, Bérénice Foncoux, Pierre Morsomme, M. Haissam Jijakli

**Affiliations:** 1 Integrated and Urban Plant Pathology Laboratory, University of Liège, Gembloux Agro-Bio Tech, 2 Passage des Déportés, 5030 Gembloux, Belgium; 2 Louvain Institute of Biomolecular Science and Technology, UCLouvain, Croix du sud 4-5, 1348 Louvain-la-Neuve, Belgium; 3 APEO SRL (Agronomical Plant Extracts & Essential Oils), Passage des Déportés 2, 5030 Gembloux, Belgium

**Keywords:** Essential oils, Weed populations, Botanical herbicides, *Arabidopsis thaliana*, Differentially accumulated proteins, Plant physiology, Proteomics, Molecular engineering in plants, Secondary metabolism, Mechanism of action, Natural products, Proteomics, Proteomics, Green chemistry

## Abstract

The increasing use of synthetic chemical herbicides has resulted in environmental, human and animal health issues. This has also led to the development of herbicide resistance in weed populations. The use of essential oils (EOs) can contribute to the development of effective, eco-friendly and nature-based alternatives to these chemical products due to their phytotoxicity and multisite action. Our study aimed to evaluate the proteomic response of *Arabidopsis thaliana* (*A. thaliana*) leaves to the application of a cinnamon essential oil (CEO) emulsion. The results showed that the application of CEO emulsion at a concentration of 6% severely impacted the proteomic profile of *A. thaliana*, especially for membrane proteins and those involved in the photosynthesis process. Interestingly, 40 proteins were identified and listed as the most differentially accumulated proteins in the leaves of *A. thaliana*. CEO decreased the expression of all the proteins associated with catabolism and anabolism processes while simultaneously increasing the expression levels of proteins involved in the response to oxidative stress. Overall, these findings allowed us to obtain a global view of the proteome response to CEO, opening promising perspectives for the development of natural herbicides, especially given the low probability of developing resistant weed populations.

**Supplementary Information:**

The online version contains supplementary material available at 10.1038/s41598-025-89368-4.

## Introduction

The intensive use of chemical pesticides is one of the major causes of biodiversity loss. It has also contributed to the development of many pesticide-resistant weed species worldwide^1^. In fact, 211 weed species have been recently identified as herbicide resistant^2^. Globally, there are 404 herbicide-resistant weed species (species × site of action). Weeds resistant to acetolactate synthase (ALS) inhibitors make up approximately one-third of all cases (133 out of 404) and are particularly problematic for rice and other cereals^3^. Unlike chemical herbicides, which have a well-defined single site of action, bioherbicides based on allelochemical molecules stand out because of their multisite actions^4^. For these reasons, the current agricultural system needs to change its practices by not only reducing the use of chemical herbicides but also using more sustainable solutions such as bioherbicides. The latter are defined as natural products used to control weeds and can be based on natural metabolites produced by living organisms, including plants and microbes^2^.

Currently, agrochemical companies are becoming increasingly interested in ecofriendly products and are investing in the research and development of biopesticides^1^. For more than three decades, the agricultural chemical sector has not introduced any new herbicides with novel sites of action, which has made farmers dependent on existing herbicides^5^. Hence, it is crucial to develop a new generation of botanical herbicides with new modes of action. In this sense, essential oils (EOs) could be among the best candidates.

EOs contain secondary metabolites produced by aromatic plants in response to biotic and abiotic stresses and provide a number of ecological advantages to plants. They contain many active compounds that are distinguished by multisite actions in plant cells, which could slow the resistance of weeds to weed killers. Another advantage of EOs is that they cause no constraint on the environment due to their high volatility and biodegradability. Moreover, EOs have shown promising herbicidal activity. Considering these factors, they constitute a good alternative to chemical herbicides^6–8^.

The phytotoxic effect of EOs on plants has been widely reported for the last 20 years. Numerous studies have shown this effect through the inhibition of seed germination and seedling growth^9–17^. For example, it has been shown that *Rosmarinus officinalis* EO, at lower concentrations, slows down the seedling growth of *Trifolium incarnatum*, *Silybum marianum*, and *Phalaris minor*, but at 5 mM, it completely inhibits seed germination^11^. This is similar to the finding^12^, who reported that *Thymbra capitata* EO inhibited the germination and seedling growth of *Erigeron canadensis L*., *Sonchus oleraceus* (L.) L., and *Chenopodium album L*. at 0.125 µL/ml.

In addition, several studies have described the site(s) of action of EOs^18–21^. These studies have shown that EOs can target the plasma membrane, cell wall, mitochondrial respiration and photosynthesis system. In fact, they can disturb the physiology and metabolic functions of weeds and lead to cell death. Nevertheless, no study has investigated the effect of EOs on the protein expression of plants.

*Cinnamomum cassia* has been traditionally used to treat gastritis and dyspepsia, blood circulation disturbances and inflammatory diseases^22,23^. Moreover, *Cinnamomum cassia* EO (CEO), like many EOs, has many medicinal and pharmacological properties, particularly antioxidant, neuroprotective, anticancer and antidiabetic properties^24–29^. It has been described in the literature that CEO has fungicidal^30–32^ bactericidal^27^, insecticidal^33–35^ and herbicidal^17,19,36^ activities, as described recently, and could be a promising alternative for chemical pesticides. To develop a better understanding of the phytotoxic effects of CEO, a label-free proteomic approach was adopted in this study to obtain a global view of the proteome response to CEO. The obtained results provide insights into the complex mode of action of CEO on *A. thaliana*.

## Methods

### Preparation of an herbicide solution based on cinnamon essential oil

CEO was purchased from Vossen & Co. (Av. Van Volxem 264/C1, 1190 Bruxelles, Belgium). The technical data sheet obtained through GC‒MS analysis revealed that the major compound was trans-cinnamaldehyde. The essential oil was formulated in water as an oil-in-water emulsion with 1% Tween 20 from Sigma‒Aldrich, which was used as a surfactant. Moreover, the concentrations of the EO were selected based on preliminary tests and literature references^17,19^.

### Postemergence test under greenhouse conditions

A postemergence experiment was conducted to study the herbicidal effect of CEO on four-week-old *A. thaliana* under controlled conditions. The greenhouse was maintained at a natural photoperiod supplemented with artificial light if needed, with temperatures set at 20 ± 3 °C according to the sunlight. The relative humidity was maintained at 60 ± 3%. Seeds of *A. thaliana* were sown in 10 × 10 cm pots (one plant per pot). The plants were watered daily to maintain adequate soil moisture and promote uniform germination and growth. Once the weeds reached the 2–3 leaf stage (after 4 weeks), two solutions were sprayed (4 mL) on leaves using small Trigger Sprayer (100 ml): (1) a negative control containing 1% Tween 20 and (2) a formulated CEO. Four replicates were considered for each condition, with each replicate containing 5 plants.

One hour after the plants were sprayed with CEO on the leaves, the plant material was collected. The second and third leaves were harvested, snap-frozen in liquid nitrogen, and stored at -80 °C. Three plants per treatment were kept to evaluate the phytotoxic effect over a 48-hour period. Additionally, after the CEO treatment, the watering remains consistent through the bottom of the pot (which has drainage holes) using a watering tray. In this case, the plants will not be affected by any water stress during the treatment. To assess the green coverage percentage during this evaluation, ImageJ software was used with the following equation:

1 $${\text{Green coverage percentage }}\left( \% \right){\text{ }} = \frac{{~{\text{green}}~{\text{surface}}~{\text{area of plant}}}}{{{\text{Total}}~{\text{surface}}~{\text{area}}~{\text{of}}~{\text{plant}}}}*100,$$

In the ImageJ analysis, the total surface area of A. *thaliana* was calculated using a broader fixed saturation range of 30 to 110, which accounts for all visible leaf tissues. This range ensures that damaged or discolored leaves are also included in the measurement. For the green surface area of the plant, this parameter represents the total area of the green parts of the leaves. In the ImageJ analysis, it was calculated using a fixed saturation range of 50 to 110, which specifically isolates the actively photosynthetic tissue. This range ensures that only the green, functional leaf areas are included in the measurement. A reduction in green surface area is an indicator of the CEO effect on the plant’s ability to photosynthesize, reflecting damage to the chlorophyll-containing tissues.

### Protein extraction

*A. thaliana* was chosen as a model plant because the full genome information was already available. For each sample, fresh matter from *A. thaliana* leaves was homogenized in a Potter homogenizer (Wheaton, IL, United States) in 800 mL of homogenization buffer (8 M urea, 100 mM TEAB (triethylammonium bicarbonate), pH 8.5 (HCl), 2 mM EDTA, 10 mM dithiothreitol (DTT), protease inhibitor mix composed of 1 mM phenylmethylsulfonyl fluoride (PMSF, Merck-Sigma-Aldrich, Darmstadt, Germany), 2 µg/mL each of leupeptin (Carl Roth, Karlsruhe, Germany), aprotinin (Carl Roth, Karlsruhe, Germany), antipain (Carl Roth, Karlsruhe, Germany), pepstatin (Carl Roth, Karlsruhe, Germany), and chymostatin (Merck-Sigma-Aldrich, Darmstadt, Germany), and 0.6% w/v polyvinylpolypyrrolidone (Polyclar^®^AT, SERVA Electrophoresis GmbH, Heidelberg, Germany). The homogenate was centrifuged for 5 min at 9000 rpm at 4 °C, and the supernatant was then centrifuged again at 54,000 rpm (TLAA55, Optima-Beckman, Indianapolis, USA) for 30 min at 4 °C. To separate the soluble and membrane fractions. Each sample after ultracentrifugation therefore gives two fractions, a supernatant called soluble fraction and a pellet called membrane fraction. The protein concentration was determined according to the Bradford method (1976) using a Bio-Rad protein assay kit with bovine gamma globulin as a standard. For each sample, 20 µg was transferred to 0.5 mL polypropylene Protein LoBind Eppendorf tubes and precipitated via the chloroform-methanol method (Wessel and Flugge 1984). To solubilize the proteins, 20 µL of 100 mM TEAB, pH 8.5 (triethylammonium bicarbonate) containing 0.5% RapiGest surfactant (Waters, Milford, USA), was added.

### In-solution trypsin digestion

The proteins were then reduced with 5 mM DTT (dithiothreitol) and alkylated with 15 mM iodoacetamide. The samples were diluted five times with 20 µL of 100 mM TEAB, pH 8.5. Proteolysis was performed with 1 µg of Sequencing Grade trypsin (Promega, Madison, USA) and was continued overnight at 37 °C. Each sample was dried under vacuum with an RVC 2–25 Martin Christ Concentrator (Martin Christ Instrument Inc., Osterode, Germany) and stored at -80 °C.

2.5 Peptide separation using nanoUPLC.

Before peptide separation, the samples were dissolved in 20 µL of 0.1% (v/v) formic acid and 2% (v/v) acetonitrile (ACN). The peptide mixture was separated by reversed-phase chromatography on a NanoACQUITY UPLC MClass system (Waters) with MassLynx V4.1 (Waters) software. For the digestion of proteins, 200 ng was injected into an ACQUITY UPLC M-Class C18 column (5 μm, 180 μm × 20 mm, 100 A) and desalted under isocratic conditions at a flow rate of 15 µL/min in 99% formic acid and 1% (v/v) ACN buffer for 3 min. The peptide mixture was subjected to reversed-phase chromatography on a C18 column (100 Å, 1.8 mm, 75 μm × 150 mm) PepMap column (Waters) for 130 min at 35 °C at a flow rate of 300 nL/min using a two-part linear gradient from 1% (v/v) ACN, 0.1% formic acid to 35% (v/v) ACN, 0.1% formic acid and from 35% (v/v) ACN, 0.1% formic acid to 85% (v/v) ACN, 0.1% formic acid. The column was re-equilibrated under initial conditions after washing for 10 min with 85% (v/v) ACN and 0.1% formic acid at a flow rate of 300 nL/min. For online LC‒MS analysis, nanoUPLC was coupled to a mass spectrometer through a nanoelectrospray ionization (nanoESI) source emitter.

### LC-IMS (Ion mobility Separation)-QTOF-MS analysis (HDMSE)

Ion mobility separation-high definition enhanced (IMS-HDMSE) analysis was performed on a SYNAPT G2-Si high-definition mass spectrometer (Waters) equipped with a NanoLockSpray dual electrospray ion source (Waters). Precut-fused silica PicoTip^R^ Emitters with outer diameters of 360 mm, inner diameters of 20 mm, 10 µm tips, and lengths of 2.5” (Waters) were used for the nanoelectrosprays. Precut-fused silica TicoTip^R^ Emitters with outer diameters of 360 mm, inner diameters of 20 mm, and lengths of 2.5” (Waters) were used for the lock mass solution. The eluent was sprayed at a spray voltage of 2.4 kV with a sampling cone voltage of 25 V and a source offset of 30 V. The source temperature was set to 80 °C. The HDMS^E^ method in resolution mode was used to collect data from 15 min to 106 min after injection. This method acquires MS^E^ in positive and resolution mode over the m/z range from 50 to 2000 with a scan time of 1 s and a collision energy ramp starting from ion mobility bin 20 (20 eV) to 110 (45 eV). The collision energy in the transfer cell for low-energy MS mode was set to 4 eV. For postacquisition lock mass correction of the data in the MS method, the doubly charged monoisotopic ion of [Glu^1^)-fibrinopeptide B was used at 100 fmol/µL using the reference sprayer of the nanoESI source with a frequency of 30 s at 0.5 ml/min into the mass spectrometer.

### ESI-QTOF data processing

HDMS^E^ data were processed with Progenesis QI (Nonlinear DYNAMICS, Waters) software using the *A. thaliana* protein sequence database (UniProt 20220410, 16127 entries). Propionamide was used as the fixed cysteine modification, oxidation was used as the variable methionine modification, trypsin was used as the digestion enzyme, and one missed cleavage was allowed. The protein confidence score is obtained by adding up the scores of all the peptides involved in the identification of the protein, even if there are some that do not participate in its quantification. The individual score of each peptide is calculated by adding up the scores obtained for a number of parameters associated with the quality of the ions detected. The tolerance on the mass and the intensities of the different isotopes must be similar. The reproducibility of the retention times and ion mobility (if used), as well as the quality of the fragmentation, should be evaluated based on the number of experimental fragments whose masses match the theoretical masses expected for a peptide (Progenesis QI, Nonlinear DYNAMICS, Waters). In addition, the mass spectrometry proteomics data have been deposited to the ProteomeXchange Consortium via the PRIDE partner repository with the dataset identifier PXD056647 and 10.6019/PXD056647.

### Statistical analysis

**Fig. 1:**
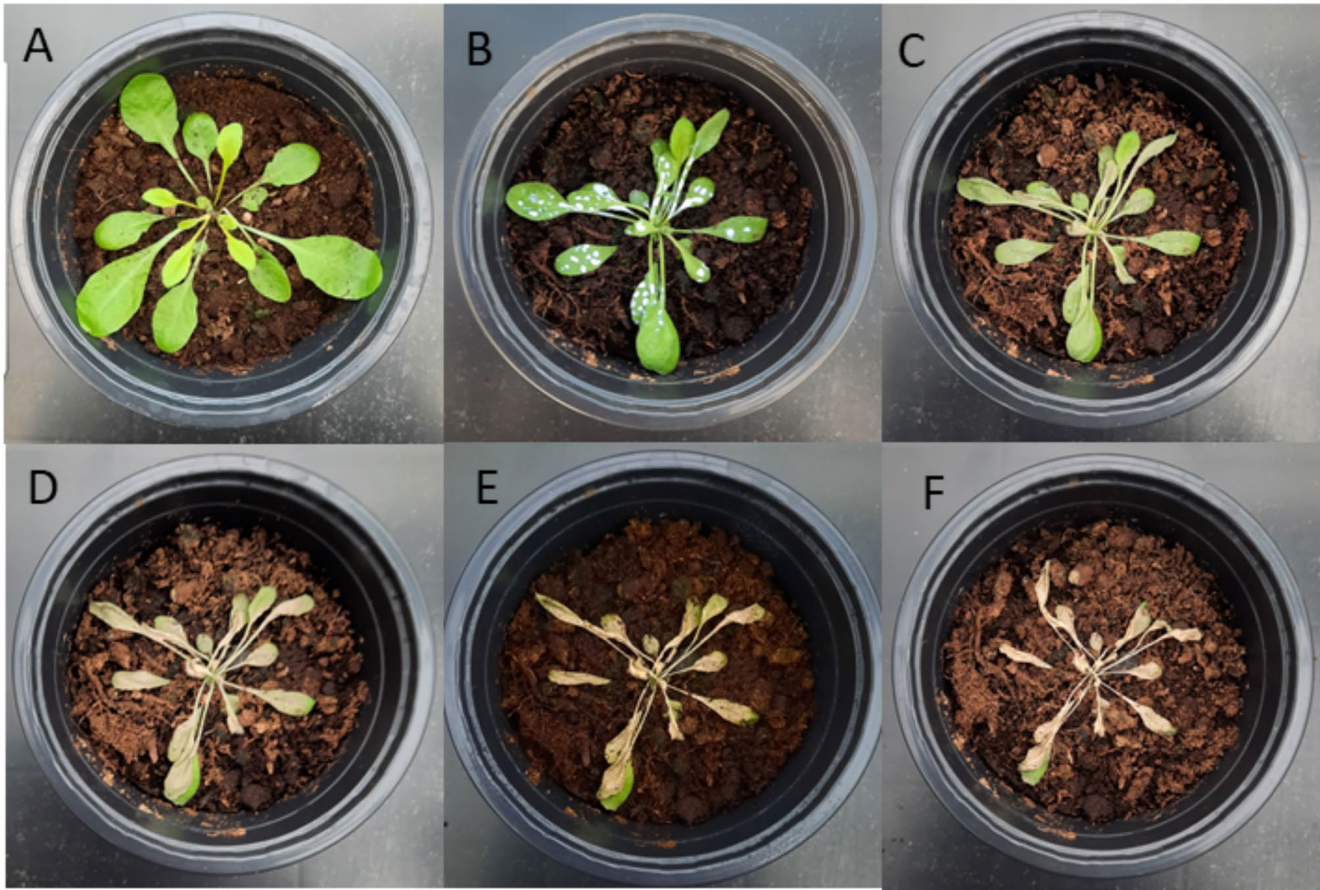
Phytotoxic effect of CEO on *A. thaliana*. (**A**) immediately prior to treatment (T0), (**B**) 5 minutes after treatment, (**C**) 1 hour after treatment, (**D**) 6 hours after treatment, (**E**) 24 hours after treatment, (**F**) 48 hours after treatment. Three plants per treatment were used for this experiment. (**B** to** F**) Show images of the same plant in the same pot at different time points after CEO treatment, illustrating the progression of the treatment’s effects over time.

Four biological replicates were used for each sample. The nonconflicting peptide method was used for relative quantification which means that proteins are quantified using only peptides that are not also part of another protein hit and protein abundance in a run is calculated from the sum of all the unique normalised peptide ion abundances corresponding to that protein (Progenesis QI, Nonlinear DYNAMICS, Waters). Statistical analyses were performed using the R (version R-4.3.0) software^37^. Protein abundances were log2-transformed and then normalized to the median. Exploratory Principal Component Analysis (PCA) was performed with missing data imputed by the regularized iterative PCA algorithm with the missMDA R package^38^. Differential abundance analyses were performed with the R package limma^39^ to compare the EO versus the control samples in each separate fraction based on moderated t-statistics. The p values were adjusted with the false discovery rate (FDR). The resulting adjusted p values and log2-fold changes are represented in the volcano plots. All tests were two-tailed. Hierarchical clustering (Euclidean distance and Ward method) and the associated heatmaps were also generated based on the z scores of proteins with adjusted p values < 0.05. To determine the differentially abundant proteins, we filtered them based on 3 criteria^40^: (1) absolute value of log2-fold change (logFC) > 1, (2) adjusted p value (adj.P. Val) < 0.05 and (3) minimum number of unique peptides *= 3* (Fig. 1; Table 1).

**Table 1:** The most affected differentially accumulated proteins presented in *A. Thaliana* treated with CEO.

Accession	logFC^1^	Fractions	Adj.*P*.Val	Peptide.count^2^	Unique.peptides^3^	Max FC	Mass	Description^4^	Function_name
AT1G52230.1	−4,023388704	Membrane	9,07502E−06	5	5	16,18499	15273,58	Photosystem I subunit H2 | Chr1:19454902–19,455,508 FORWARD LENGTH = 145 | 201,606	Photosynthesis
AT2G34420.1	−3,768434308	Membrane	0,000176642	12	5	15,53079	28,111	Photosystem II light harvesting complex protein B1B2 | Chr2:14522716–14,523,513 REVERSE LENGTH = 265 | 201,606	Photosynthesis
AT1G37130.1	−2,971004071	Soluble	1,78668E−06	7	3	7,957331	103756,9	Nitrate reductase 2 | Chr1:14158617–14,161,652 FORWARD LENGTH = 917 | 201,606	Anabolism process
AT4G10340.1	−2,953029549	Membrane	2,55066E−06	37	27	8,179058	30213,75	Light harvesting complex of photosystem II 5 | Chr4:6408200–6,409,496 FORWARD LENGTH = 280 | 201,606	Photosynthesis
AT5G23890.1	−2,916107939	Membrane	1,11007E−06	14	4	8,232063	104212,2	GPI-anchored adhesin-like protein | Chr5:8058789–8,063,005 FORWARD LENGTH = 946 | 201,606	Photosynthesis
AT1G17600.1	2,794157541	Membrane	3,61966E−05	9	3	6,077531	120229,5	Disease resistance protein (TIR-NBS-LRR class) family | Chr1:6053026–6,056,572 REVERSE LENGTH = 1049 | 201,606	response to stress/signalization
AT1G01100.1	−2,773114309	Membrane	2,54096E−06	5	3	12,33421	11218,73	60 S acidic ribosomal protein family | Chr1:50284–50,954 REVERSE LENGTH = 112 | 201,606	response to stress/signalization
AT5G12860.1	−2,690044501	Membrane	8,13004E−07	8	7	6,972963	59498,39	Dicarboxylate transporter 1 | Chr5:4059927–4,061,919 REVERSE LENGTH = 557 | 201,606	response to stress/signalization
AT5G64290.1	−2,670330083	Membrane	1,11007E−06	5	4	6,866718	60333,75	Dicarboxylate transport 2.1 | Chr5:25714495–25,716,642 REVERSE LENGTH = 563 | 201,606	response to stress/signalization
AT4G26690.1	−2,63317636	Membrane	2,03962E−06	9	5	6,733917	83018,32	PLC-like phosphodiesterase family protein | Chr4:13456793–13,459,890 REVERSE LENGTH = 759 | 201,606	response to stress/signalization
AT1G32080.1	−2,602482586	Membrane	1,11447E−05	8	6	6,802451	54530,15	Membrane protein | Chr1:11537572–11,539,756 REVERSE LENGTH = 512 | 201,606	response to stress/signalization
AT3G48110.1	2,574154918	Membrane	5,76413E−06	12	6	5,417182	120229,7	|Glycine-tRNA ligase | Chr3:17763111–17,770,964 FORWARD LENGTH = 1067 | 201,606	Anabolism process
AT2G29630.1	2,47890068	Membrane	0,000766622	6	3	5,403282	72614,1	ThiaminC | Chr2:12667395–12,669,569 FORWARD LENGTH = 644 | 201,606	Anabolism process
AT4G35790.1	−2,475790486	Membrane	1,29353E−06	10	6	6,088241	99658,51	Phospholipase D delta | Chr4:16955774–16,959,875 REVERSE LENGTH = 868 | 201,606	Catabolism process
AT4G18480.1	2,472842745	Membrane	2,11819E−06	17	5	5,090073	46555,12	P-loop containing nucleoside triphosphate hydrolases superfamily protein | Chr4:10201897–10,203,361 REVERSE LENGTH = 424 | 201,606	Photosynthesis
ATCG00720.1	−2,442813931	Membrane	1,33082E−06	10	9	9,323731	24266,68	Photosynthetic electron transfer B | ChrC:74,841–76,292 FORWARD LENGTH = 215 | 201,606	Photosynthesis
AT4G27520.1	−2,435682215	Membrane	1,41094E−06	10	6	5,87544	35178,32	Early nodulin-like protein 2 | Chr4:13750668–13,751,819 REVERSE LENGTH = 349 | 201,606	Unkonwn
AT4G25450.1	−2,421336431	Membrane	1,79023E−06	8	5	5,862783	78496,75	Nonintrinsic ABC protein 8 | Chr4:13009845–13,013,912 REVERSE LENGTH = 714 | 201,606	Anabolism process
AT2G40100.1	−2,386903471	Membrane	5,99979E−06	7	6	5,573684	30268,64	Light harvesting complex photosystem II | Chr2:16745884–16,747,190 FORWARD LENGTH = 276 | 201,606	Photosynthesis
AT5G46800.1	−2,365199358	Membrane	5,61959E−07	6	3	5,594747	31193,89	Mitochondrial substrate carrier family protein | Chr5:18988779–18,989,810 REVERSE LENGTH = 300 | 201,606	Catabolism process
AT1G12900.1	2,352475411	Membrane	2,8648E−07	34	18	5,005567	43132,02	Glyceraldehyde 3-phosphate dehydrogenase A subunit 2 | Chr1:4392634–4,394,283 REVERSE LENGTH = 399 | 201,606	Catabolism process
AT4G22240.1	−2,334282194	Soluble	0,000115282	5	3	4,966773	33712,48	Plastid-lipid associated protein PAP/fibrillin family protein | Chr4:11766090–11,767,227 REVERSE LENGTH = 310 | 201,606	response to stress/signalization
AT2G39730.1	−2,097433323	Soluble	9,09128E−06	42	32	4,362756	52380,53	Rubisco activase | Chr2:16570951–16,573,345 REVERSE LENGTH = 474 | 201,606	Anabolism process
AT4G25080.1	−1,995141413	Soluble	1,67894E−06	9	3	4,128942	33966,91	Magnesium-protoporphyrin IX methyltransferase | Chr4:12877015–12,878,128 FORWARD LENGTH = 312 | 201,606	Anabolism process
AT4G27520.1	1,944816924	Soluble	1,68778E−06	9	6	3,718391	35178,32	Early nodulin-like protein 2 | Chr4:13750668–13,751,819 REVERSE LENGTH = 349 | 201,606	Anabolism process
AT2G45470.1	1,9308956	Soluble	1,68778E−06	8	4	3,660294	43188,92	FASCICLIN-like arabinogalactan protein 8 | Chr2:18742797–18,744,059 REVERSE LENGTH = 420 | 201,606	response to stress/signalization
AT1G54780.1	1,8125804	Soluble	2,22868E−06	7	5	6,287133	31138,64	| Thylakoid lumen 18.3 kDa protein | Chr1:20439533–20,440,953 FORWARD LENGTH = 285 | 201,606	Photosynthesis
AT5G13030.1	−1,799722131	Soluble	3,92451E−06	5	3	3,608215	71839,38	| selenoprotein O | Chr5:4133216–4,136,461 FORWARD LENGTH = 633 | 201,606	response to stress/signalization
AT1G12900.1	−1,794018002	Soluble	2,50935E−05	28	17	3,528933	43132,02	Glyceraldehyde 3-phosphate dehydrogenase A subunit 2 | Chr1:4392634–4,394,283 REVERSE LENGTH = 399 | 201,606	Catabolism process
AT5G14040.1	−1,793712191	Soluble	0,000597415	8	3	3,680165	40488,85	Phosphate transporter 3%3B1 | Chr5:4531059–4,532,965 REVERSE LENGTH = 375 | 201,606	response to stress/signalization
AT5G65010.1	−1,793602652	Soluble	6,97154E−06	4	3	3,57252	65600,25	Asparagine synthetase 2 | Chr5:25969224–25,972,278 FORWARD LENGTH = 578 | 201,606	Anabolism process
AT3G46970.1	−1,759871356	Soluble	1,68778E−06	14	9	3,51415	95558,59	Alpha-glucan phosphorylase 2 | Chr3:17301625–17,306,111 REVERSE LENGTH = 841 | 201,606	Catabolism process
AT4G17300.1	−1,722265077	Soluble	1,74122E−05	4	3	3,366175	64211,64	Class II aminoacyl-tRNA and biotin synthetases superfamily protein | Chr4:9681558–9,684,833 FORWARD LENGTH = 567 | 201,606	Anabolism process
AT3G44110.1	−1,664909946	Soluble	4,5875E−06	5	3	3,286595	47071,65	DNAJ homolog 3 | Chr3:15869115–15,871,059 REVERSE LENGTH = 420 | 201,606	protein folding
AT1G42970.1	−1,623904278	Soluble	1,21097E−05	29	18	3,157349	48115,85	Glyceraldehyde-3-phosphate dehydrogenase B subunit | Chr1:16127552–16,129,584 FORWARD LENGTH = 447 | 201,606	Cellular respiration
AT5G28840.1	−1,617578974	Soluble	1,68778E−06	9	7	3,174705	43157,79	GDP-D-mannose 3’%2C5’-epimerase | Chr5:10862472–10,864,024 REVERSE LENGTH = 377 | 201,606	Anabolism process
AT3G48000.1	1,607736297	Soluble	1,68778E−06	17	7	5,254714	58988,2	Aldehyde dehydrogenase 2B4 | Chr3:17717082–17,719,843 REVERSE LENGTH = 538 | 201,606	response to stress/signalization
AT3G60130.1	−1,579476771	Soluble	6,97154E−06	6	3	3,100083	58442,03	Beta glucosidase 16 | Chr3:22210343–22,213,650 FORWARD LENGTH = 514 | 201,606	Catabolism process
AT5G50920.1	−1,574157919	Soluble	6,0626E−06	37	3	3,082209	103680,8	CLPC homolog 1 | Chr5:20715710–20,719,800 REVERSE LENGTH = 929 | 201,606	Structure organization
AT4G27430.1	1,551503373	Soluble	2,50935E−05	7	3	2,797201	118652,9	COP1-interacting protein 7 | Chr4:13718817–13,722,736 FORWARD LENGTH = 1058 | 201,606	Anabolism process

^1^ Fold change.

^2^ The number of peptides relative to proteins.

^3^ the number of peptides identified in the sample related to the protein.

^4^ Based on tair data base.

In addition, the identified differentially abundant proteins were annotated and grouped by function bins based on the MapMan ontology for *A. thaliana* downloaded from GoMapMan^41^. They are represented in a heatmap with the average abundance per group of samples for each functional bin. This average abundance is calculated by taking the mean value of log normalized abundances where the grand mean has been added.

## Results

### Overview of the proteome profile of leaves treated with CEO

**Fig. 2:**
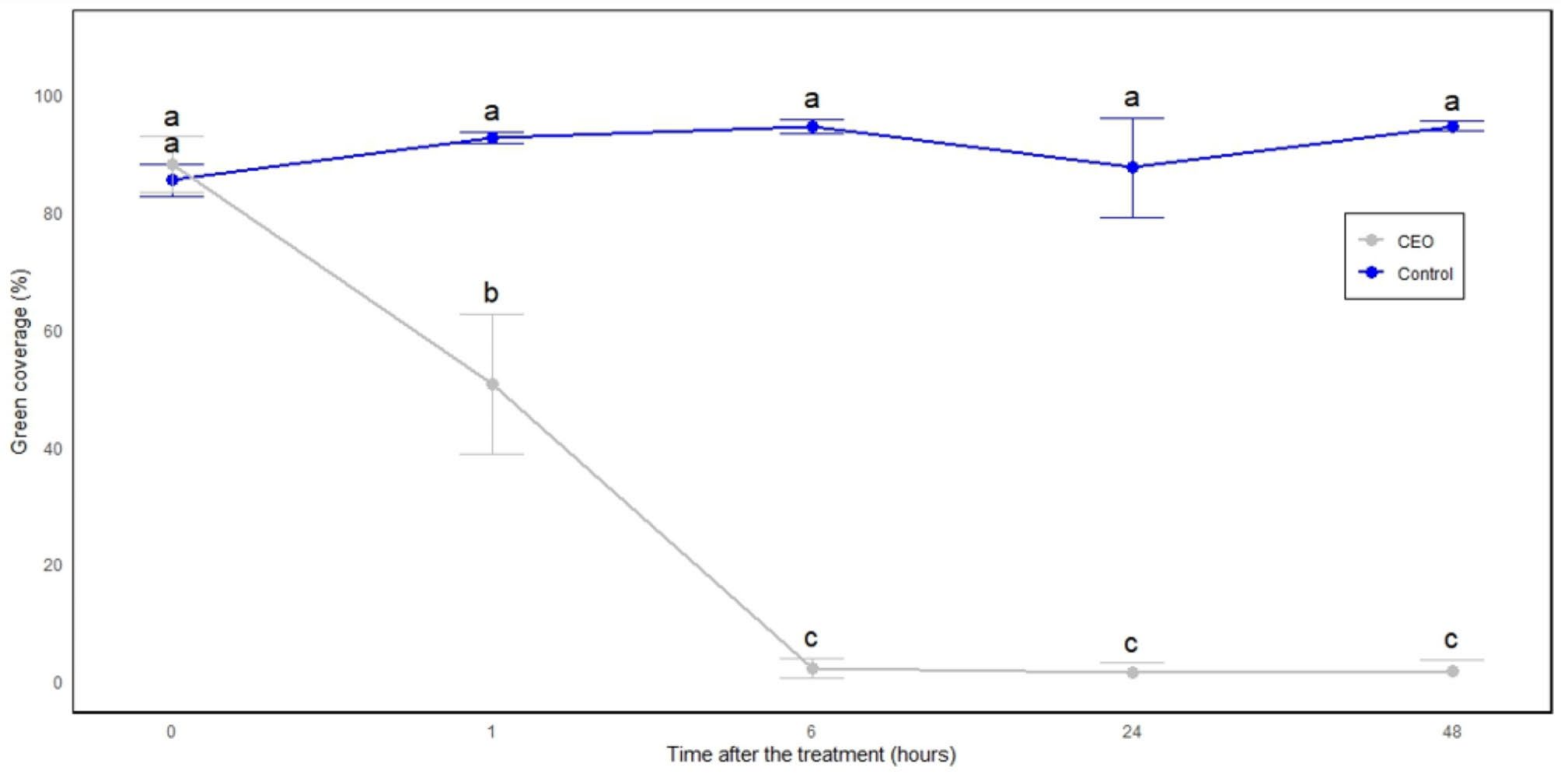
Green coverage percentage of *A. thaliana* treated with CEO at 6%. Results were statistically analyzed using one-way analysis of variance (ANOVA, R software) followed by Tukey’s multiple range test. Differences between individual means were considered significant at *p* < 0.05. Therefore, values in a figure followed by the same letter are not significantly different. Statistical analysis was performed based on the “time of treatment” rather than the treatment itself. ImageJ software was used to evaluate the green coverage percentage during this assessment, as detailed in the Materials and Methods section.

A phenotypic experiment was conducted to assess the phytotoxic effects of CEO over a 48-hour period. As shown in Figs. 1 and 2, the herbicidal activity was observed on *A. thaliana*. Leaf wilting occurred just 1 h after treatment, with a 42.53% reduction in green coverage (Fig. 1C), followed by a noticeable decrease in green pigmentation after 6 h (Fig. 1D). By 48 h, both the leaves and stems were completely withered and discolored, reaching a 98.1% reduction in green coverage (Fig. 1F).

**Fig. 3:**
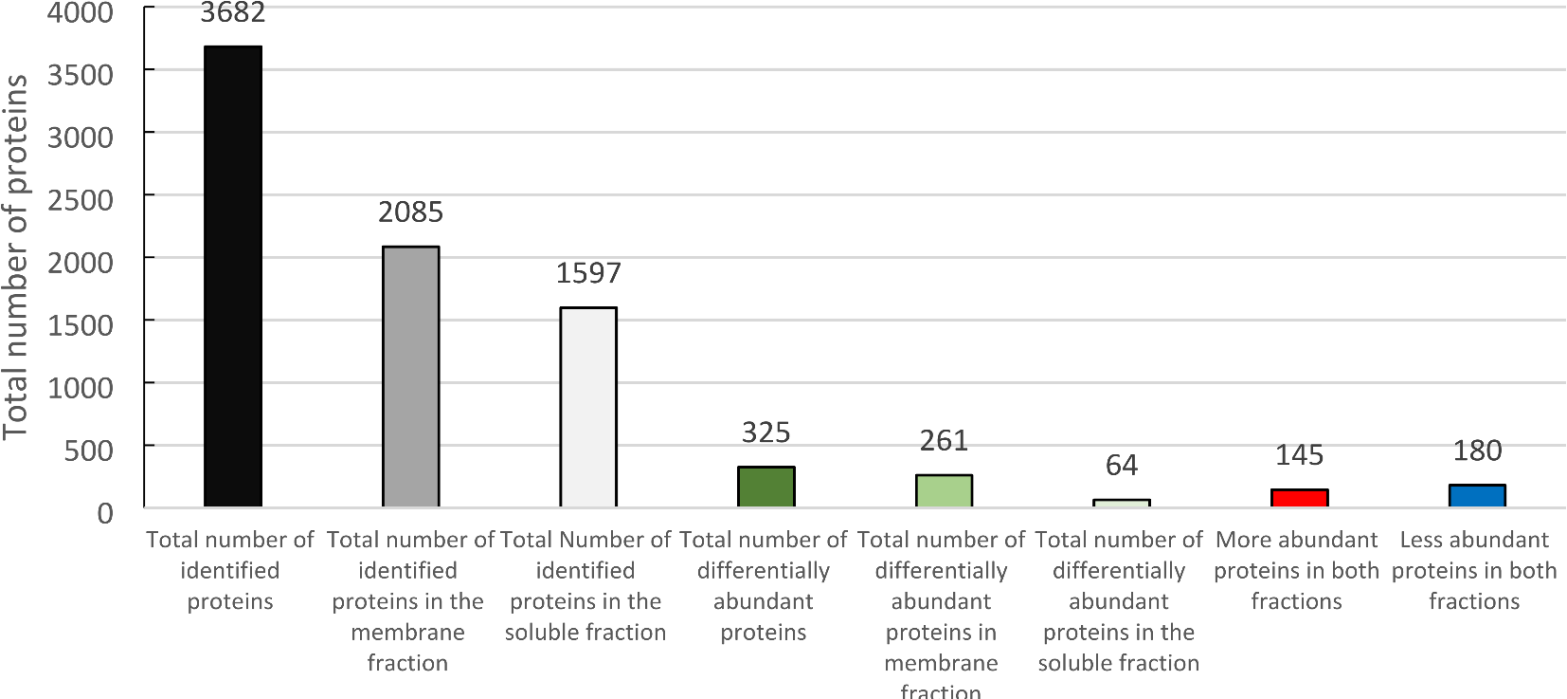
Number of proteins identified and differentially accumulated in leaves of *A. thaliana* treated with CEO. The number of abundant proteins was determined by filtering the proteins based on 3 criteria: (1) absolute value of log2-fold change (logFC) > 1, (2) adjusted p value (adj.P. Val) < 0.05 and (3) minimum number of unique peptides = 3.

Then, we used a label-free quantitative proteomics approach to investigate the herbicidal effect of CEO. In total, 3682 proteins were identified and quantified in *A. thaliana* leaves. All the sequences of the identified peptide fragments in the soluble and membrane fractions can be found in supplementary Table S1 and Table S2, respectively. Compared to those in untreated leaves, 325 differentially accumulated proteins were found, and among them, 145 were overaccumulated, while 180 were downregulated (Fig. 3).

**Fig. 4:**
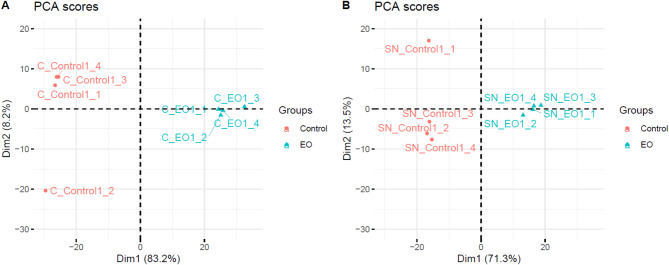
Score plot of the principal component analysis of all identified proteins of *A. thaliana* in the membrane fraction. (**A**) Membrane fraction (**B**) Soluble fraction.

The scores plot of the PCA represented in Fig. 4 clearly distinguishes between the control and treated leaves of *A. thaliana* by CEO. Indeed, the first PCA dimension, representing the main axis of variation, enables the separation of the controls from the EOs both in the soluble fraction (71.3%) and in the membrane fraction (83.2%).

**Fig. 5:**
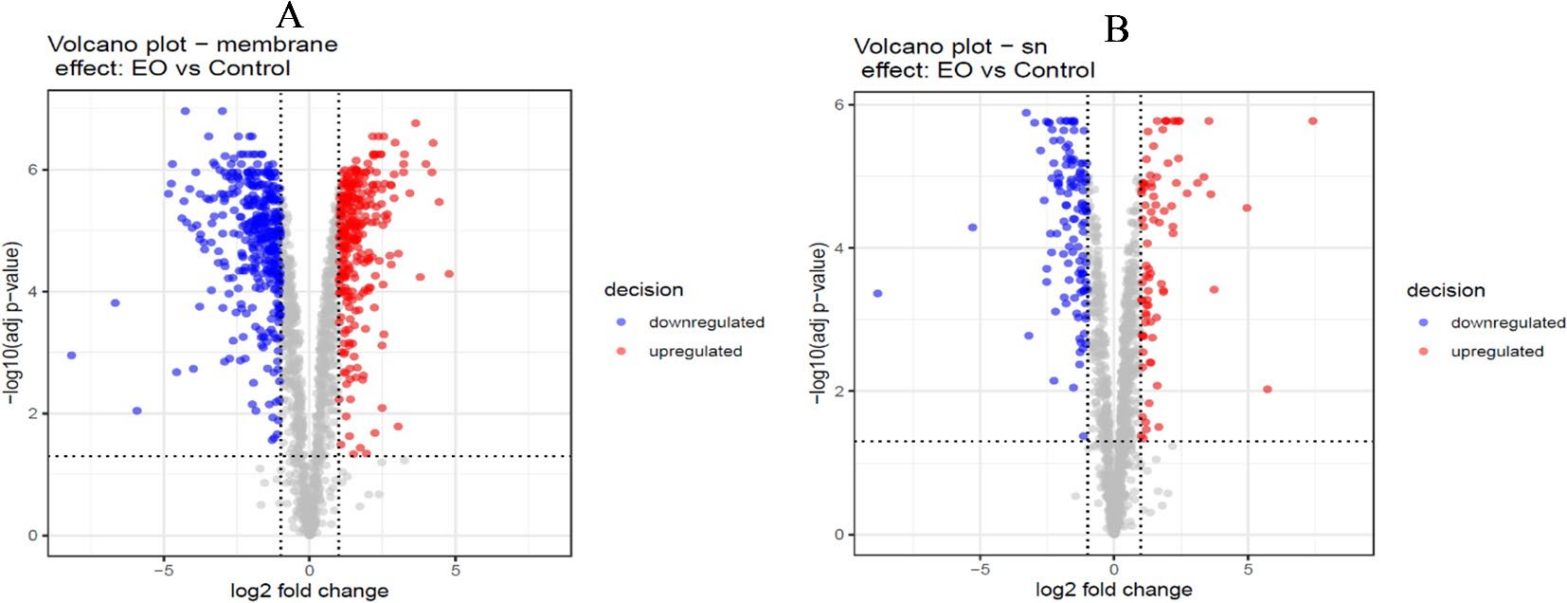
Volcano plot analysis showing differentially accumulated proteins between control leaves of *A. thaliana* and leaves treated with CEO. (**A**) membrane fraction (adjusted p value < 0.05 and absolute log fold change > 1), (**B**) soluble fraction (adjusted p value < 0.05 and absolute log fold change > 1). Blue dots represent underaccumulated proteins, and red dots represent overaccumulated proteins; gray dots are not significantly up- or downregulated.

The volcano plots illustrate and link log fold changes and adjusted p values for each protein (Fig. 5A and B). It showed that a high number of differential accumulated protein were identified after only 1 h of treatment with CEO.

**Fig. 6:**
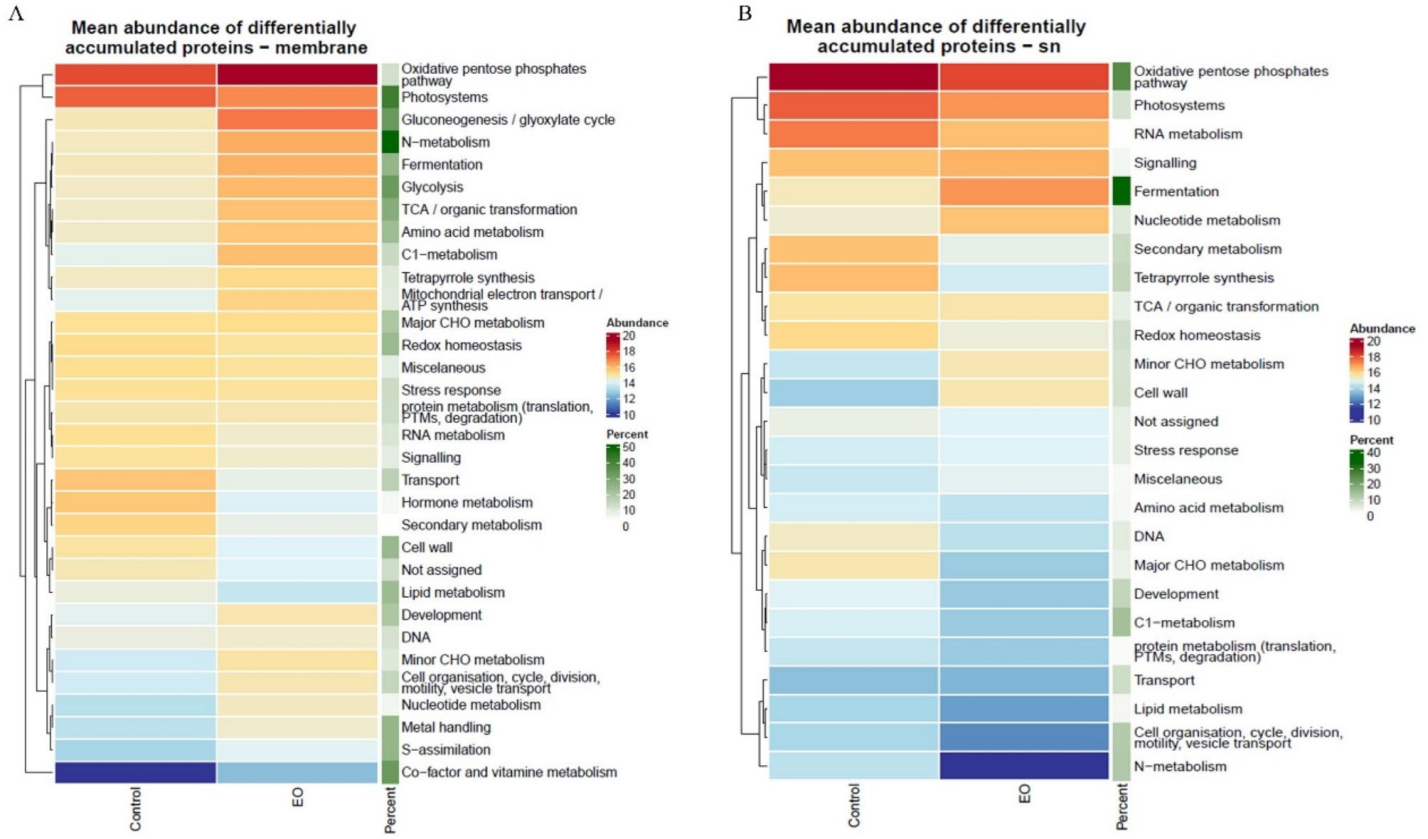
Heatmap analysis showing the average abundance of all differentially accumulated proteins (adjusted p value < 0.05 and absolute log fold change > 1 and minimum number of unique peptides = 3) between control leaves of *A. thaliana* and leaves treated with CEO, gathered into MapMan functional bins. This average abundance is calculated by taking the mean value of log normalized abundances where the grand mean has been added. The “percent” column represents the proportion of quantified proteins belonging to a functional bin that are identified as differentially abundant proteins. A: membrane fraction, B: soluble fraction.

To further analyze the data, we performed heatmap clustering, which highlighted significant proteomic changes across various functional categories, as represented by the MapMan functional bins (Fig. 6A, and 6B). The differentially accumulated proteins were classified into 31 categories for the membrane fraction and 25 categories for the soluble fraction. It revealed significant proteome remodeling after CEO exposure, impacting an essential metabolic pathway such as the pentose phosphate cycle, glycolysis, photosynthesis, nitrogen metabolism, fermentation, and cell wall synthesis. These changes confirm the substantial damage caused by CEO. In addition, among the most affected differentially accumulated proteins, we identified the photosystem II subunit H2 light-harvesting complex protein, photosystem I subunit H2 protein, GPI-anchored adhesin-like protein, and nitrate reductase 2 with the highest fold changes, which reached 16.18, 15.53 8.23 and 7.96, respectively (Table 1). Indeed, proteins involved in metabolic processes were also differentially accumulated (e.g., phospholipase D delta glyceraldehyde 3-phosphate dehydrogenase asparagine synthetase 2 alpha-glucan phosphorylase 2). All these modifications certainly impacted the accumulation of proteins involved in the response to oxidative stress as well as proteins involved in the transduction of cellular signals (e.g., dicarboxylate transporter 1, PLC-like phosphodiesterase family protein plastid-lipid associated protein PAP selenoprotein O).

## Discussion

In this study, we found that 6% CEO caused wilting of leaves after only one hour of spraying, which confirmed its phytotoxic effect and highlights its contact herbicidal actions. This effect has been demonstrated for the first time by Tworkoski, 2002 ^36^, who showed the strong phytotoxic activity of CEO against *Chenopodium album*, *Ambrosia artemisiifolia*, and *Sorghum halepense*. He found that 2% of CEO caused rapid leaf injury and strong electrolyte leakage. In addition to its herbicidal activity, CEO has several other biological properties. In this context, the insecticidal activity of CEO against *Sitophilus zeamais* on maize was reported^33^ and the fungicidal activity of the same EO against *Botrytis cinerea* on pears after 4 days has also been described^42^. All studies confirmed that cinnamaldehyde, the lead compound, is responsible for the toxic effect of CEO. Furthermore, the phytotoxic effect of phenylpropanoids, including cinnamaldehyde, on the leaves of *A. thaliana* could be explained by their interaction with membrane receptors, unlike monoterpenes, which disturb lipid organization^19^. It is crucial to remember that the toxic properties of essential oils depend strongly on their chemical composition, which is affected by genetic variation, sampled plant tissues, growing conditions and extraction methods^11^. In the case of herbicidal activity, another factor was the tested weed species. In fact, phytotoxic activity can be more effective on one plant species than on another. For instance, the foliar application of a Caraway EO emulsion had a greater impact on the biochemical parameters of barnyard grass than on those of maize^43^. On this subject, the selective action of an EO toward one undesired plant species and not another is due to the mode(s) of action of its compounds, which tend to block one metabolic pathway in some plants and not others^44^.

In this paper, we studied the herbicidal effect of CEO on *A. thaliana* through protein expression. To our knowledge, this is the first time that label-free quantitative proteomic technology has been used to analyze the biochemical responses of plants after treatment with EOs. This advanced analytical method has been used to create cellular proteome maps and characterize interactions between plants and pathogens or defense reactions to biotic or abiotic stress^45^. It has also been used to facilitate comparative and proteomic analyses of complex samples. Nevertheless, it will be necessary in the future to improve separation technology and bioinformatic analysis.

Our proteomic analysis revealed that CEO induced dramatic changes in the leaves of *A. thaliana* after only one hour of exposure. Indeed, 325 proteins were differentially accumulated between the treated leaves and untreated leaves. A similar study was conducted to investigate the insecticidal effect of *Mentha arvensis* essential oil on the weevil of *Sitophilus granarie*^40^. A total of 55 differentially accumulated proteins were detected. They showed that after 24 h of contact, the toxicity of this essential oil to insects had a notable impact on various biological processes, especially those related to the nervous and muscular systems. Due to their abundance of active compounds, essential oils offer a multitude of mechanisms of action with a low probability of developing resistant weed populations^46^. This will be further discussed below.

Among the 27 herbicide groups, 7 directly disturb the photosynthesis system of weeds by inhibiting key enzymes, especially 4-hydroxyphenylpyruvate dioxygenase (HPPD inhibitors). They can also bind to protein complexes present in the chloroplast thylakoid membrane and consequently completely stop the electron transport chain, as is the case for triazine. It has been described in the literature that photosynthesis is one of the most important biological processes in plant physiology, allowing the production of oxygen and energy in the form of sugar^47^. Several studies have confirmed that photosynthesis is inhibited in the presence of allelochemicals, particularly EOs^2,21,48^. A phenotypic experiment showed that by 48 hours, the stems and leaves became discolored and dried, confirming the desiccant effect caused by the disruption of photosynthetic mechanisms. This is further supported by our proteomic analysis, which revealed that just one hour was enough to completely destabilize photosynthetic activity in *A. thaliana*, as evidenced by a significant reduction in photosystem proteins in both the soluble and membrane fractions. On the other hand, proteomic analyses revealed additional differentially accumulated proteins that were not associated with the visual observations, such as nitrate reductase, which plays a key role in nitrogen assimilation in plants. This is illustrated in Fig. 6, which shows that 31 physiological processes in *A.thaliana* were disrupted. Among these processes, nitrogen metabolism, pentose phosphate pathway, along with fermentation, were significantly affected. These results could be directly in line with Ben kaab et al., 2020^48^, who state that plant extracts containing multiple molecules usually exhibit multisite action, which contrasts with synthetic herbicides that typically target a single site. It has also been shown that EO decreases water content and consequently acts as a desiccant herbicide^11,59^. This could be supported by our proteomic analysis, which showed overexpression of some proteins involved in the retention of water content in plants through the regulation of stomatal closure (phospholipase D delta protein and membrane protein AT1G32080.1). In addition, photosystem subunit 1 protein, photosystem 2 light-harvesting protein and photosynthetic electron transfer B protein are integral components of the four protein complexes located on the thylakoid membrane of the chloroplast which are strongly downregulated (Table 1). They play an important role in the preservation of the electrochemical gradient required for the phosphorylation of ADP to ATP^47^. The rubisco-activated protein was also downregulated. This protein is absolutely necessary for photosynthesis, particularly because it allows for the fixation of atmospheric CO_2_ and its subsequent incorporation in the Calvin cycle for energy production in the form of sugar^49^. The thylakoid lumen protein, which is also one of the top 40 DEPs, was downregulated. It maintains photosystem 2 under high light and contributes to the phosphorylation of ADP to ATP by pumping H + to the stroma^50^. All these results are in agreement with those of Li et al., 2021^51,^ who confirmed that the phytotoxic effect of essential oils is related to the inhibition of *A. thaliana* photosynthesis. They revealed for the first time the possibility that the essential oil of *Artemisia argyi* may act as an HPPD inhibitor to block the photosynthesis chain in weed species. In fact, they analyzed the HPPD content by an immunosorbent assay (ELISA) kit and showed that at 4 µL/mL, the HPPD content significantly decreased by 31.24% in comparison to that in the control group. In addition, herbicides that specifically inhibit HPPD, such as mesotrione, effectively manage a broad spectrum of weed species^52^. It is important to mention that the main compounds of *Artemisia argyi* essential oil are monoterpenes that can thus penetrate the cell and damage cellular organelles without affecting membrane permeability due to their small size^53^.

The plasma membrane serves as a solid barrier separating the cell from its surroundings and plays a vital role in the perception of external signals, facilitating exchanges between the cytoplasm and the cellular environment^54,55^. Thus, any alteration in the structure of the plant plasma membrane caused by bioactive compounds will disrupt its function and integrity and consequently disturb biochemical and physiological processes^56,57^. For these reasons, scientists believe that the plant plasma membrane is one of the potential cellular targets of essential oils (EOs). The authors also suggested first studying the interactions between phytochemical compounds and the plasma membrane to understand the mode of action of these compounds^58–60^. These compounds can interact with lipid membranes and can react as pro-oxidants by inducing lipid peroxidation^48^. Molecular dynamic simulations revealed that cinnamaldehyde (CIN) molecules can penetrate only up to the polar head region of the model plasma membrane, where they can interact with membrane proteins, such as membrane receptors and ion channels^19^. This finding is in line with the results of our study. In fact, CEO downregulated 264 protein membranes in *A. thaliana* leaves. These membrane proteins are present not only in the plasma membrane but also in various cellular organelles, including the thylakoids of chloroplasts, mitochondria, the endoplasmic reticulum, the Golgi apparatus, lysosomes and peroxisomes. This made it challenging to identify the specific proteins of the plasma membrane.

As shown in Fig. 6, CEO affect the secondary metabolism and the signalization process. Importantly, all types of constraints on plants induce oxidative stress^61^. In addition, allelochemical compounds can induce oxidative stress by generating reactive oxygen species (ROS). The latter are highly reactive, which can make them toxic in certain cases^62^. They play an important signaling role in regulating essential processes such as growth, development, response to biotic and abiotic environmental stimuli, defense against pathogens and stomatal behavior^63^. ROS can react directly with biological molecules, such as DNA, proteins or lipids, generating mutations and damaging membranes, leading to cell and tissue damage and causing programmed cell death (PCD) processes^64^. The main type of ROS is O_2_, which can be transformed into another harmful ROS, such as a hydroxyl radical. Excessive ROS production also causes oxidative damage to cellular proteins, lipids, and nucleic acids and activates death pathways in several cell types^65^.

To summarize the mechanism of action of CEO, phenotypic evidence demonstrates its rapid effect, consistent with its contact effect on the leaf cuticle. This effect is further confirmed by the downregulation of proteins involved in the biosynthesis of the cuticle, as shown by proteomic analysis. Furthermore, the observed leaf discoloration and drying validate its desiccant properties. Proteomic analysis supports this observation, as overexpression of certain proteins involved in water retention has been noted. This could be related to a decrease in membrane integrity, as shown by Ben Kaab et al. 2020^48^, leading to water leakage. It is also well known that essential oils contain small molecules that are able to easily interact with the plant cell membranes, which could induce a prooxidant effect, commonly referred to as the “burndown effect.” This was confirmed by researchers at the WSSA Annual Meeting, who affirmed that most plant-based bioherbicides produce burning effects^66^ Consequently, we observed an overexpression of proteins involved in managing oxidative stress, resulting from oxidative damage to membrane systems on one hand, and, on the other hand, the desiccant effect of CEO, which results from the loss of membrane integrity. This will likely change the expression of several proteins, particularly those involved in photosynthesis and fermentation, which is highly dependent on water.

Concerning the label-free protein quantification method, it requires high reproducibility in sample preparation and handling^67,68^. Variations may occur between samples analyzed by LC/MS even between technical replicates. Unlike labeling quantification methods where all samples are analyzed together, it is necessary to analyze all samples separately. This therefore requires processing a large number of replicates to obtain statistically stable data. Normalizing a large number of replicates can ultimately reduce the number of proteins of interest^69^. If some proteins are too abundant, less expressed proteins will be less well or not at all identified.

## Conclusion

Currently, there is a significant demand for more research to develop natural products for agronomic application. Unfortunately, the authorization processes in EU states are time-consuming, complex and expensive and require safety documentation, such as for ecotoxicological studies. Understanding the mode(s) of action is crucial for conducting these studies efficiently. Interestingly, our research confirmed that cinnamon essential oil (CEO) could be a promising botanical herbicide for controlling weed invasion. This has been confirmed by its high and rapid phytotoxicity. Notably, our proteomic approach showed, for the first time, that a high number of proteins could be differentially accumulated after only one hour of CEO treatment. The results also showed that photosynthesis was strongly inhibited by the reduction in the expression of photosystem proteins in thylakoid membranes. It is also important to mention that quantification by the label-free approach offers a greater dynamic range and broader identified protein coverage, but lower quantification accuracy and reproducibility^70^. Finally, this study showed that CEO has a strong herbicidal effect, making it a suitable source of natural herbicides with a low probability of developing resistant weed populations. In future studies, Other weed and crops types should be tested to better understand the herbicidal effects of CEO. Additionally, field trials should be conducted to evaluate this activity under uncontrolled conditions. Since CEO has contact herbicidal action, studying its effect on the cuticle and cell wall is crucial for determining its mode of action.

## Electronic supplementary material

Below is the link to the electronic supplementary material.

Supplementary Material 1

Supplementary Material 2

## Data Availability

The mass spectrometry proteomics data have been deposited to the ProteomeXchange Consortium via the PRIDE partner repository with the dataset identifier PXD056647 and 10.6019/PXD056647.
